# Prediabetes Burden in Nigeria: A Systematic Review and Meta-Analysis

**DOI:** 10.3389/fpubh.2021.762429

**Published:** 2021-12-23

**Authors:** Musa Ado Bashir, Anas Ibrahim Yahaya, Mukhtar Muhammad, Ashiru Hassan Yusuf, Isyaku Gwarzo Mukhtar

**Affiliations:** ^1^Department of Anatomy, Bayero University Kano, Kano, Nigeria; ^2^Department of Physiology, Bayero University Kano, Kano, Nigeria

**Keywords:** prediabetes, Nigeria, impaired glucose tolerance, systematic review, meta-analysis

## Abstract

Prediabetes is a borderline glycemic status associated with both higher incidence of cardiovascular disease as well as higher risk of progression to diabetes. There is a rising burden of diabetes and prediabetes globally. This study aims to estimate the burden of prediabetes in Nigeria. Online searches of Google Scholar, PubMed, and Scopus were conducted and studies were selected based on predefined criteria. A total of 15 studies consisting of 14,206 individuals conducted between 2000 and 2019 were included in the meta-analysis with studies using American Diabetic Association (ADA) and World Health Organization (WHO) criteria pooled separately. The pooled prevalence of prediabetes in Nigeria was found to be 13.2% (95% CI: 5.6–23.2%, *I*^2^ = 98.4%) using the ADA criteria and 10.4% (95% CI: 4.3–18.9%, *I*^2^ = 99.2%) using the WHO criteria. According to the latest data by the United Nations, this translates to an estimated 15.8 and 12.5 million adult prediabetic individuals in Nigeria using the ADA and WHO criteria, respectively. The prevalence rates for women and men were similar at 12.1% (95% CI: 5–21%). The pooled prevalence rates for urban and rural settlements were also similar at 9% (95% CI: 2–22%). In conclusion, the prevalence of prediabetes in Nigeria was almost two times higher than the 7.3% estimate by the International Diabetes Federation in 2003. The similar rates of prediabetes between men and women and between urban and rural settlements points toward narrowing of cardiovascular risk burden between the two sexes and the two settlements. This represents higher future cardiovascular disease burden in the country further pressurizing the overstretched healthcare system.

## Introduction

Prediabetes is a borderline glycemic recognized as a toxic cardio-metabolic state similar to what is observed in established diabetes (1). Consequently, many macrovascular and microvascular complications of diabetes exist in the prediabetic state (2). Prediabetes is a heterogenous entity including impaired fasting glucose (IFG), defined as fasting blood sugar (FBS) in the range 5.6–6.9 mmol/L, impaired glucose tolerance (IGT), defined as 2-h blood sugar between 7.8 and 11.0 mmol/L during a glucose tolerance test (OGTT), and raised glycosylated hemoglobin (HbA1c) levels in the range 5.7–6.4% (3).

A recent meta-analysis of 59 prospective studies, showed that pre-diabetic individuals have almost six times the risk of developing diabetes than normoglycemic individuals. This risk ranged from as high as eleven times for studies that used the HbA1C or ADA FBS criteria to as low as three times for studies that used IGT criteria (4). Prediabetes not only predisposes individuals to higher risk of progression to diabetes but is by itself an independent risk factor for cardiovascular disease. A meta-analysis of 129 studies, comprising 10,069,955 individuals, found that prediabetes predisposes patients to higher risk of all cause mortality, composite cardiovascular disease, coronary heart disease, and stroke. In particular, the WHO FBS criteria of prediabetes were associated with a 1.26 relative risk of all cause mortality compared to normoglycemia. The corresponding risk for ADA FBS criteria was 1.03. The corresponding relative risks for coronary heart disease for the two criteria were 1.12 and 1.05, respectively. Interestingly, the meta-analysis reported a lower relative risk of developing stroke, compared to normoglycemia, if ADA FBS criteria were used. The WHO FBS criteria, however, were associated with a stroke relative risk of 1.18 compared to normoglycemia (5).

In another recent meta-analysis of 15 prospective studies with a median follow-up of 8 years and comprising 9,827,430 individuals, prediabetes was associated with increased incidence of heart failure (RR 1.58, 95% CI 1.04–2.39, for IGT) compared to normoglycemia (6). Prediabetes was also found in another recent meta-analysis to adversely affect outcome in heart failure patients with an all cause mortality HR of 1.29 (95% CI 1.06–1.58) (7).

The International Diabetes Federation (IDF) estimated the prevalence of prediabetes in Nigeria in 2003 to be at 7.3% and projected that, by 2025, the number of individuals with prediabetes in the country will almost double but the prevalence will remain at 7.3% because of the increasing population (8). A more recent regional estimate sets the prevalence of prediabetes (defined as IGT) of the African region at 10.1% (CI = 5.6–22.7%) translating to 45.3 million individuals. It is of note that neither the 2003 nor the 2019 IDF reports included data from Nigeria in the estimation of prediabetes prevalence (8, 9). To the best of our knowledge, this is the first systematic review and meta-analysis of prediabetes prevalence in Nigeria since the 2003 publication by the IDF.

## Methodology

### Study Area

**Figure 1:**
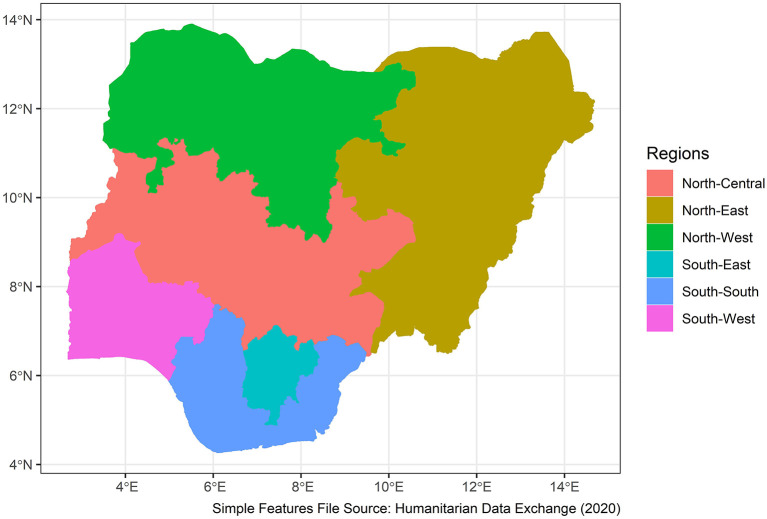
Geo-political zones in Nigeria.

Nigeria is a western African nation with an area of 923,769 sq km, home to more than 250 ethnic groups (10). It has 36 states and a capital divided into 6 geo-political zones or regions. The estimated population in 2021 is 211.4 million. A total of 43.4% of the population are under the age of 14 years. Overall, 53.9% of the population are between the ages of 15 and 64 years. Only 2.8% of the population are above the age of 65 years (11). Figure 1 shows the map of the geopolitical zones of the country.

### Inclusion and Exclusion Criteria

We included community-based studies conducted in adult population (>18 years of age) of Nigeria. Studies selected defined prediabetes using both ADA and WHO criteria and used HbA1C, FBS, and IGT definitions. We excluded studies conducted in individuals <18 years of age, studies on pregnant women, and all hospital-based studies.

### Studies Search Strategies

The online databases Google Scholar, PubMed, and Scopus were used. The search words and phrases “impaired fasting glucose,” “impaired fasting sugar,” “impaired glucose tolerance,” “prediabetes,” and “borderline glycemic state” were used. The search was repeated for each word or phrase with the name “Nigeria.” The search was conducted between March 2021 to November 2021. Screening of the abstracts and titles of the articles was done independently by two reviewers (MAB and IGM), and studies selection and exclusion were completed based on the predefined criteria. Thereafter, screening of the full-text articles was done independently by the same researchers to select the studies to be included in qualitative and quantitative analyses.

### Qualitative Analysis of the Included Studies

The methodological quality of the included studies was assessed using a modification of the Joanna Briggs Institute (JBI) Critical Appraisal Checklist for Studies Reporting Prevalence Data (12). The item's original nine questions were reduced to six with questions 1 and 2 given a score of 2 and the remaining four questions each given a score of 1 for a “yes” and 0 for a “no.” The total maximum score is 8. A study was judged as good quality if it scored a minimum of 6 and of poor quality if it scored <6. Assessment was done independently by two reviewers (AHY and MM) with disagreements sorted by AIH. Table 1 shows the modified tool used in critical appraisal of the included studies. The minimum sample size for scoring a study as a “yes” was 102 calculated using Epitools' (13) online calculator assuming an estimated prevalence of 7.1% based on a recent meta-analysis of studies conducted in neighboring Cameroon (14). Reliability and validity of methods were assessed based on whether the studies used glucose oxidase methods or point-of-care glucometers for measurement of blood glucose levels. Only studies judged as having high methodological quality were included in the quantitative analysis.

**Table 1:** Critical appraisal checklist.

**S.N**	**Question**	**Yes**	**No**
1	Was the sample frame appropriate to address the target population?	2	0
2	Were study participants sampled in an appropriate way?	2	0
3	Was the sample size adequate?	1	0
4	Were the study subjects and the setting described in detail?	1	0
5	Were valid methods used for the identification of the condition?	1	0
6	Was the condition measured in a standard, reliable way for all participants?	1	0
	Total Score		

### Data Extraction and Quantitative Analysis

Data extraction was independently done by two reviewers (MAB and IGM). Extracted information from the studies included prevalence of prediabetes, sample size, settlement (urban/rural), state and region of the study, study year, mean age, and sex composition of the study participants. Data were entered into Excel and then imported into R statistical environment for statistical computing, version 4.1.0.1 (15). The meta for package (16) was used to fit the multi-level random effects model for pooling prevalence rates and the multi-level mixed effects model for meta-regression using the inverse variance method with correction of pooled estimate and its variance using Sidik-Jonkman's estimator for between-study heterogeneity (17). The three levels of the multi-level models are as follows:

1. Level 1: variance explained by sampling errors of the included studies
2. Level 2: between-study heterogeneity
3. Level 3: heterogeneity between clusters of studies with clusters defined by the methods of defining prediabetes, i.e., IGT, HbA1c, FBS under ADA criteria, and FBS under WHO criteria.

Because the ADA and IGT criteria for prediabetes are not mutually exclusive (both defined IGT the same way), comparison of the pooled prevalence rates under the two criteria could not be done through meta-regression or sub-group analyses. Consequently, the prevalence rates under the two criteria were pooled separately.

The analysis of heterogeneity was done both through a meta-regression using characteristics of the included studies as predictors. Comparison of between-study heterogeneity measures in the model with and model without moderators was done. Additionally, distribution of heterogeneity between the three levels of the model was calculated using the formula developed by Cheung (18) and implemented in the dmetar R package (19). Finally, prediction intervals were reported to overcome the difficulties in interpreting both tau^2^ and *I*^2^ as measures of between-study heterogeneity (20).

A funnel plot was used to visually inspect for possible publication bias where studies reporting small prevalence were not published and thus not included in the meta-analysis. The formal regression test developed by Egger and colleagues (21) was employed for testing funnel plot asymmetry.

## Results

### Search Results

**Figure 2:**
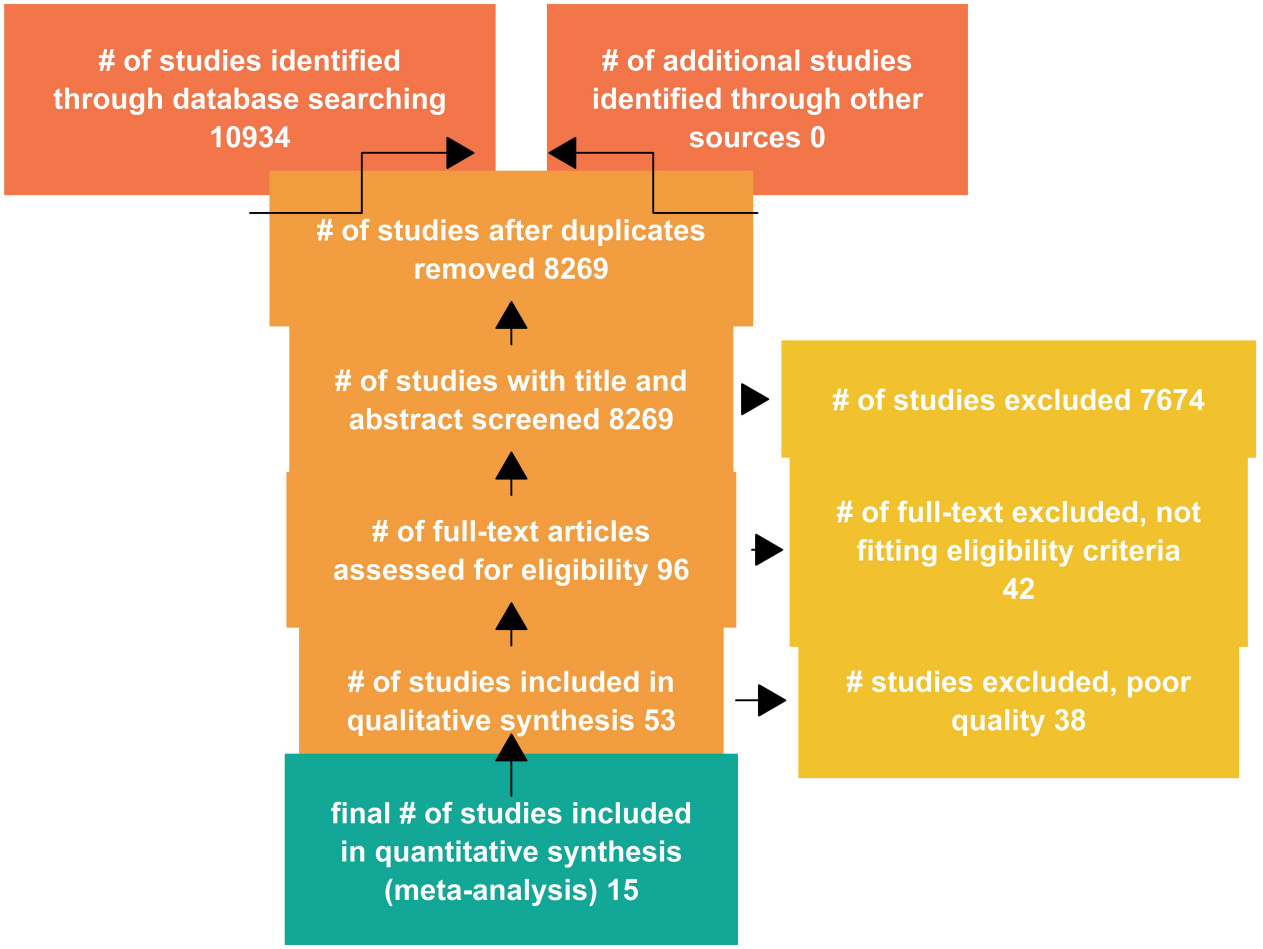
Results of the search strategy.

A total of 10,934 studies were retrieved from the databases, with titles and abstracts of 8,269 studies screened after duplicates were removed. The full text of 96 studies were assessed for eligibility, and a total of 53 studies were included in qualitative analysis (Figure 2).

### Methodological Features and the Critical Appraisal of the Included Studies

Overall, 53 studies were selected and included in the qualitative analysis of methodological features. The quality appraisal of the included studies is shown in Supplementary Table 1.

### Characteristics of the Studies Included in the Quantitative Analysis

A total of 15 studies consisting of 14,206 individuals met the inclusion criteria and were included in the meta-analysis. The studies were conducted between 2000 and 2019. There were four studies from the north-west region of which two were conducted in urban settlements, one was conducted in a rural area, and one study was conducted in both rural and urban settlements. For the latter study, data on prevalence of prediabetes in rural and urban areas were analyzed separately. There were two studies from the north-central area both conducted in urban settlements. There were two studies from the south-east conducted in an urban and a rural settlement. Four studies were conducted in the south-south region including three studies conducted in urban settlements and one study conducted in a rural settlement. For the latter study, data on prevalence of prediabetes in rural and urban areas were analyzed separately. Two studies were conducted in the south-west region in an urban and a rural settlement. No study from the north-east region was included in the quantitative analysis. Overall, four studies were only conducted in the rural settlements, nine studies were only conducted in urban settlements, and two studies were conducted in both rural and urban settlements.

**Figure 3:**
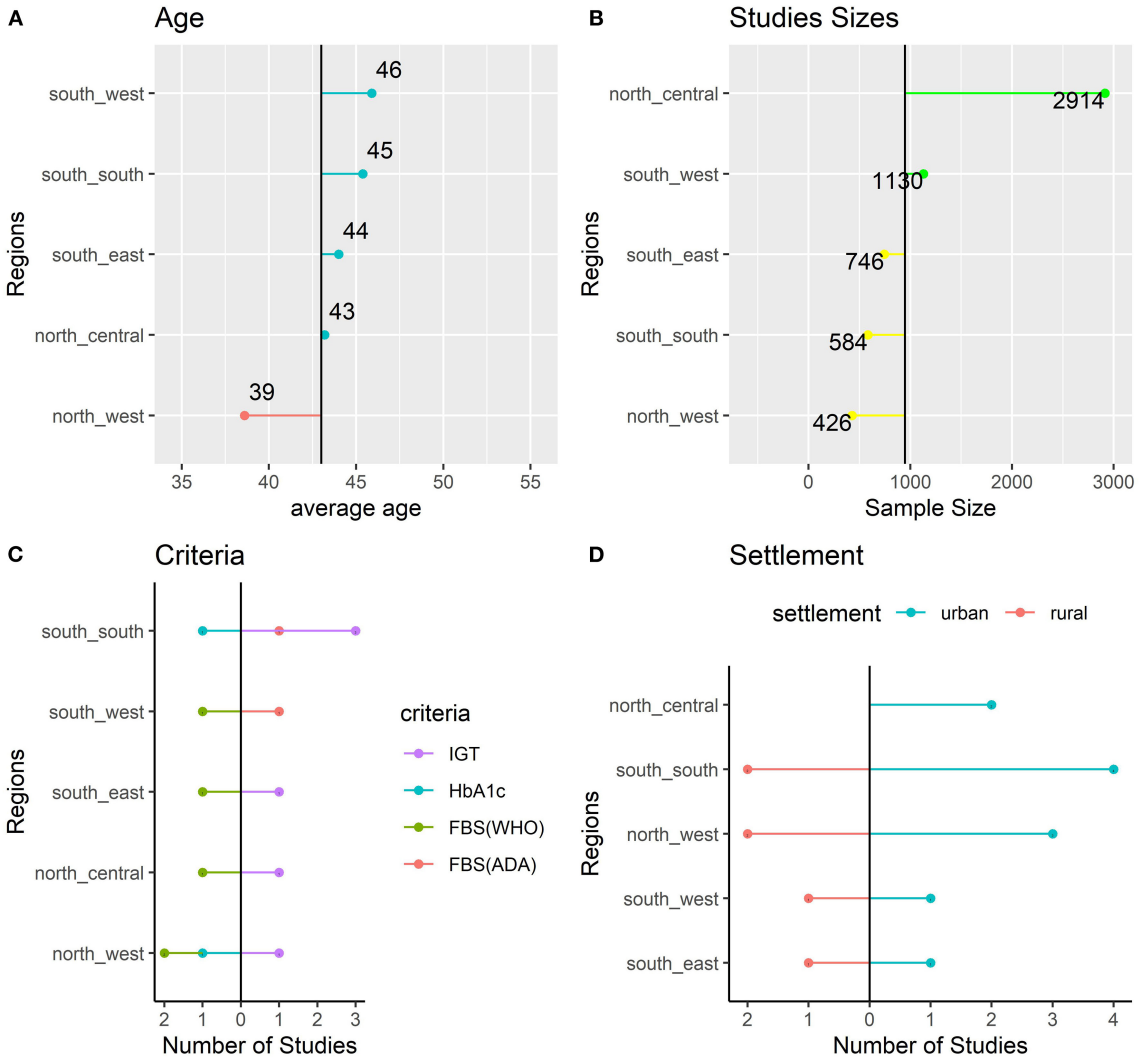
Characteristics of the Included Studies: age **(A)**, sample size **(B)**, criteria **(C)**, and settlement **(D)**.

Impaired glucose tolerance (IGT) was the most frequent criteria used by the included studies with six studies using the criteria. Two studies used the HbA1c criteria and six (6) studies used the FBS criteria. The region with the youngest study participants was the north-west with a reported mean age of 39. South-west studies had the oldest participants with a mean age of 46. Studies conducted in the southern regions included older subjects than those included in northern regions (Figure 3).

### Fitting the Meta Analytic Model

**Figure 4:**
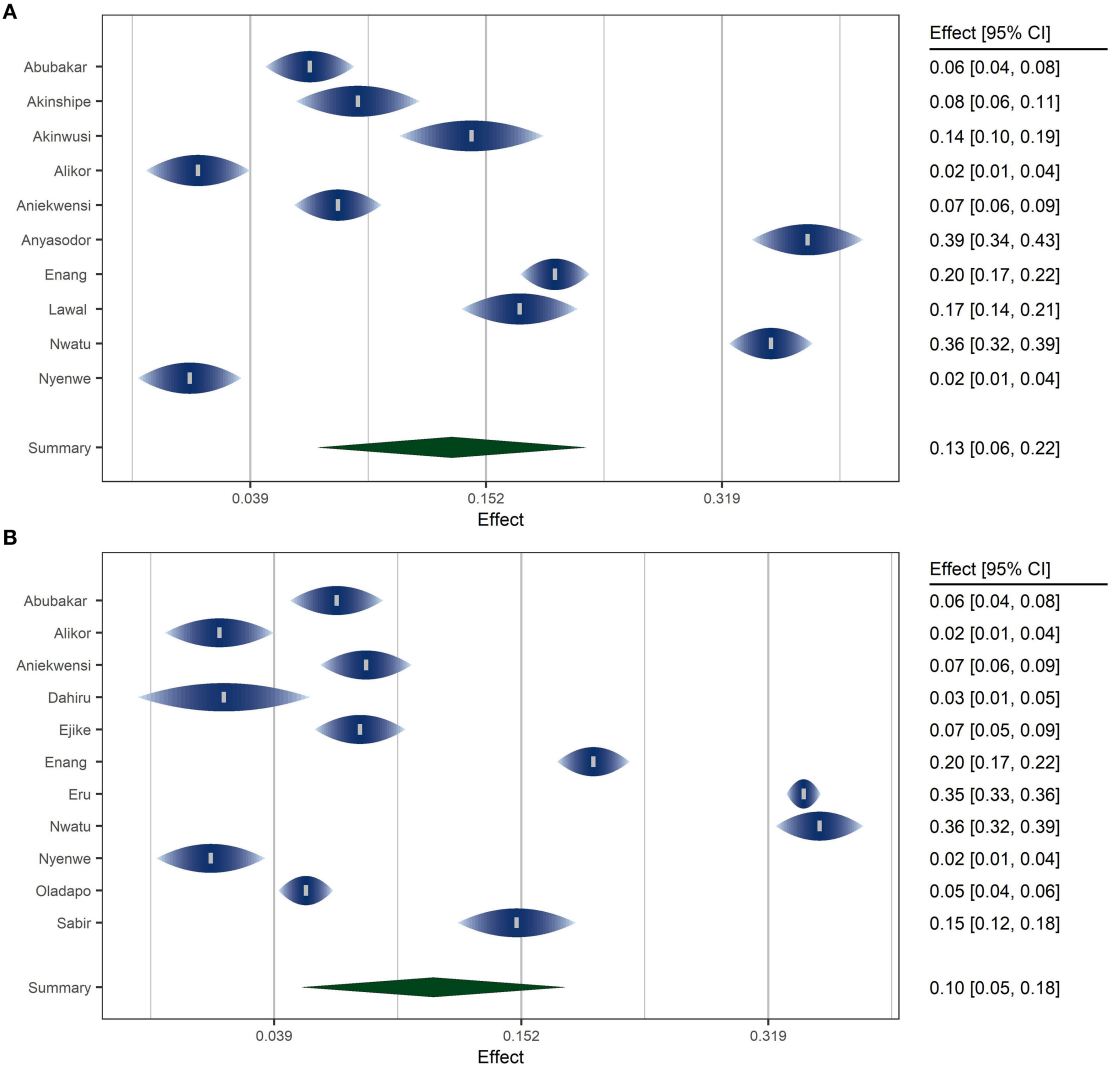
Forest plots of Studies using ADA [plot **(A)**] and WHO [plot **(B)**] Criteria.

A random effects model was fitted using the inverse variance method with correction of pooled estimate and its variance using Sidik-Jonkman's estimator for between-study heterogeneity. Prevalence rates were transformed using arcsine transformation. Prevalence rates under ADA and WHO criteria were pooled separately. Figure 4 shows the forest plots of the model.

The overall pooled prevalence of prediabetes in Nigeria was 13.2% (95% CI: 5.6–23.2%) using the ADA criteria and 10.4% (95% CI: 4.3–18.9%) using the WHO criteria. According to the latest data by the United Nations (11), this translates to an estimated 15.8 million and 12.5 million adult prediabetic individuals in Nigeria using the ADA and WHO criteria, respectively. The *P* values for the random meta analytic model was <0.001 for the models under the two criteria (ADA and WHO). The estimated total between-study heterogeneity not explained by sampling error (*I*^2^) under both criteria was about 99.2%. The test for heterogeneity is significant with a *p* < 0.001 indicating substantial heterogeneity between the included studies. Prediction intervals for the models under ADA and WHO criteria were 0–51.8 and 0–46%, respectively.

### Analysis of Between-Study Heterogeneity

**Figure 5:**
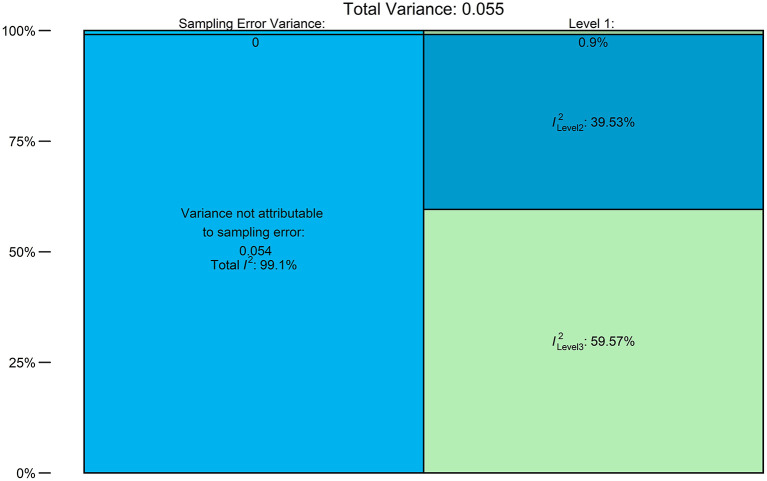
Distribution of Heterogeneity: Level 1 = sampling error, level 2 = within-study heterogeneity, and level 3 = between-criteria heterogeneity.

For the model under ADA criteria, a multilevel meta-regression model was fitted using the geo-political region as a moderator. Figure 5 shows the distribution of the heterogeneity. Most of the substantial heterogeneity (almost 60% of it) was attributable to the use of different methods of defining prediabetes by the included studies (IGT, HbA1c, or FBS). The between-study heterogeneity (level 2 of the model) accounted for about 40% of the heterogeneity.

**Figure 6:**
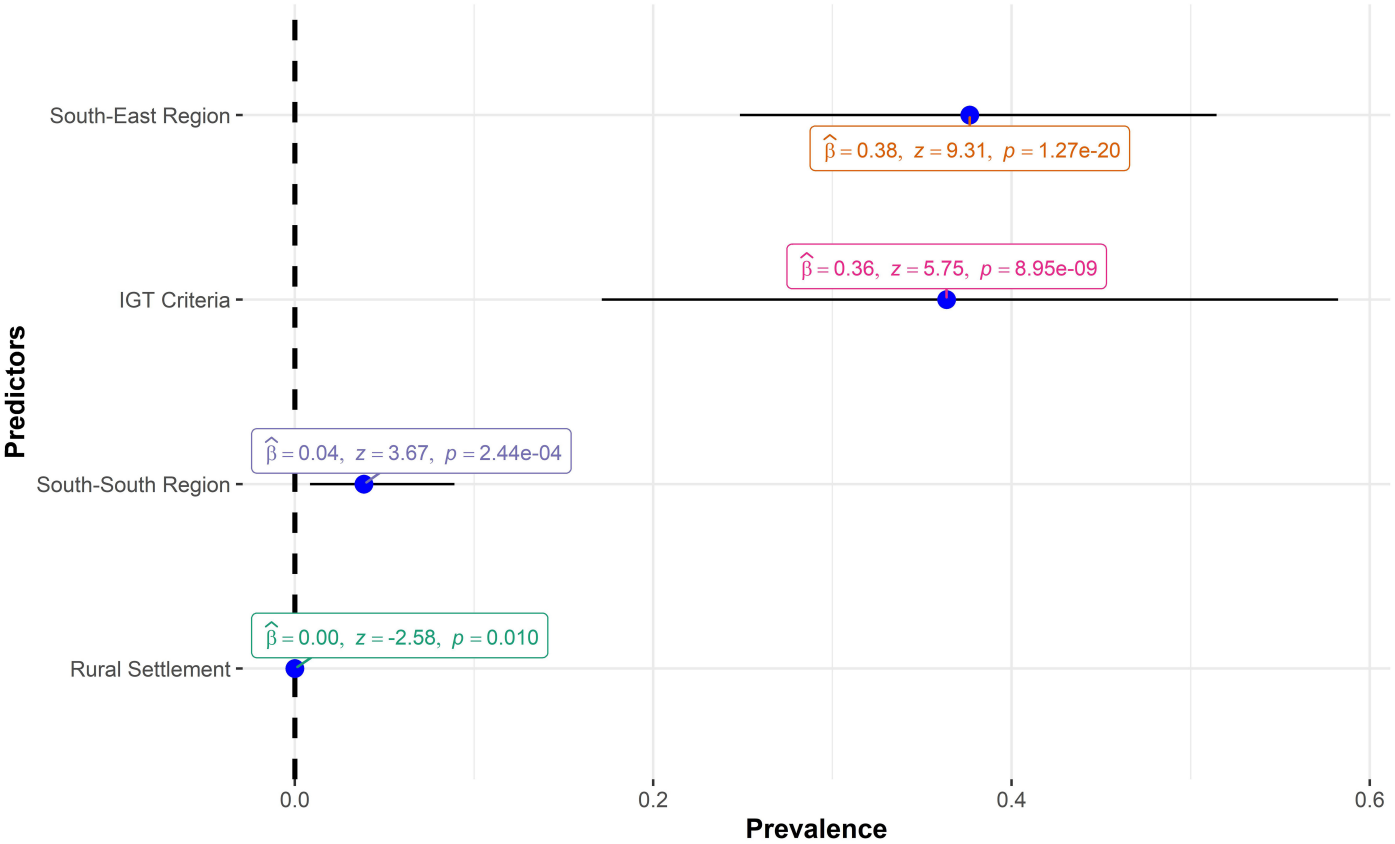
Regression coefficients of the WHO criteria meta-regression.

For the WHO criteria model, a meta-regression model using gender, region, and mean age of the participants as moderators was fitted. The value of *I*^2^ dropped from 99.2%, signifying substantial heterogeneity, to 50.1% indicating moderate heterogeneity with a statistically non-significant test of between-study heterogeneity (*P* = 0.217). This means most of the heterogeneity between the studies resulted from the differences in study characteristics. Figure 6 shows the statistically significant regression coefficients, in decreasing order, of the predictors under WHO criteria.

### Analysis of Publication Bias

**Figure 7:**
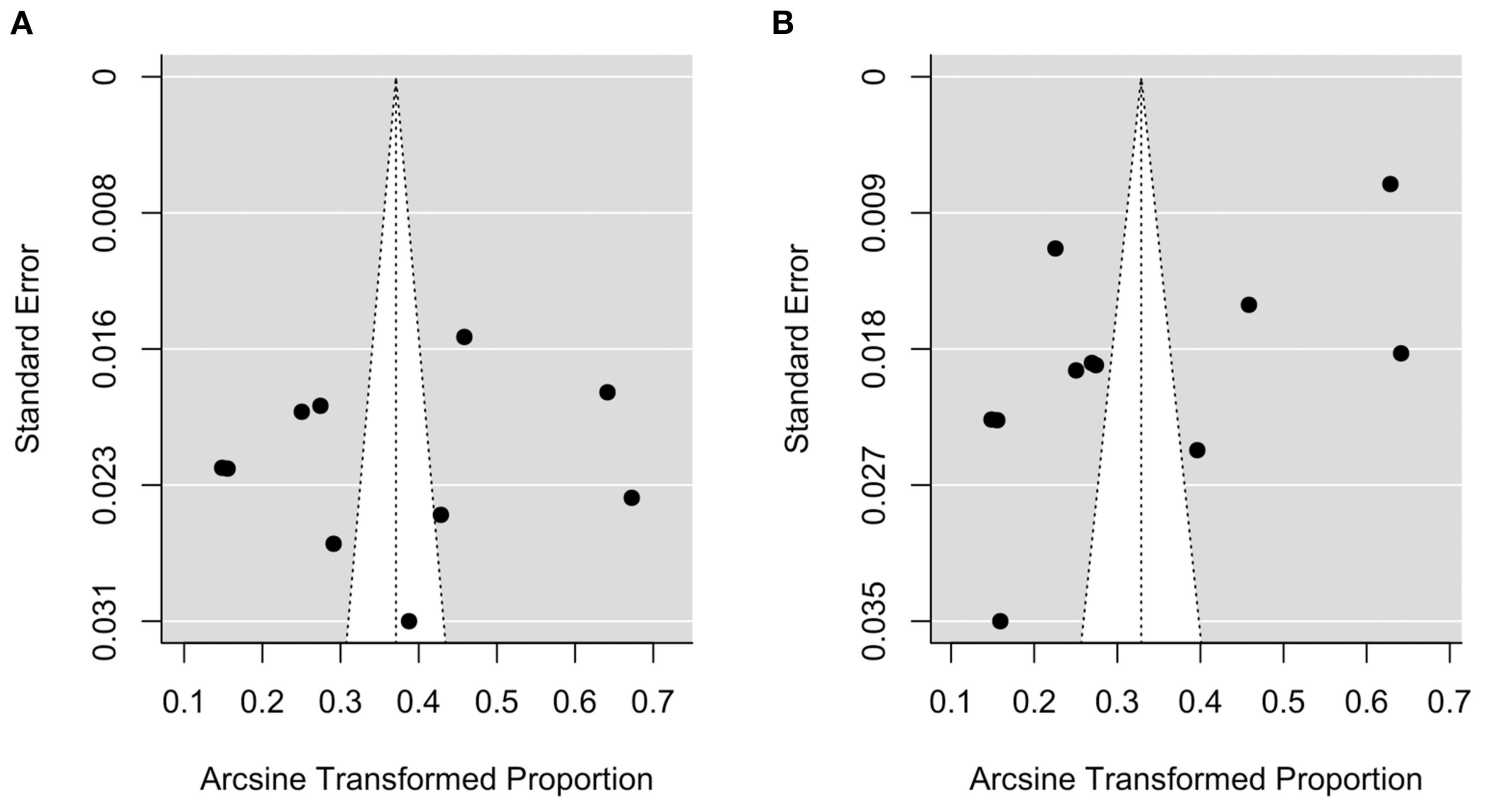
Funnel plots of the ADA [plot **(A)**] and WHO [plot **(B)**] criteria models.

Figure 7 shows the funnel plot of the model under ADA [plot (A)] and WHO [plot (B)] criteria, respectively. There was no obvious asymmetry in the plots. A formal test for plot asymmetry (regression test) was conducted and it was not statistically significant (*P* value was 0.816 and 0.052 for the model under ADA and WHO criteria, respectively), confirming the visual assessment of the funnel plot.

### Gender-Specific Prevalence of Prediabetes

The prevalence rates for women and men were similar at 12.1% (95% CI: 5–21%) and 10.4% (95% CI: 4–20%) under WHO and ADA criteria, respectively.

### Prevalence of Prediabetes Among Urban and Rural Settlements in Nigeria

The pooled prevalence rates for urban and rural settlements were similar at 9% (95% CI: 2–22%).

## Discussion

**Figure 8:**
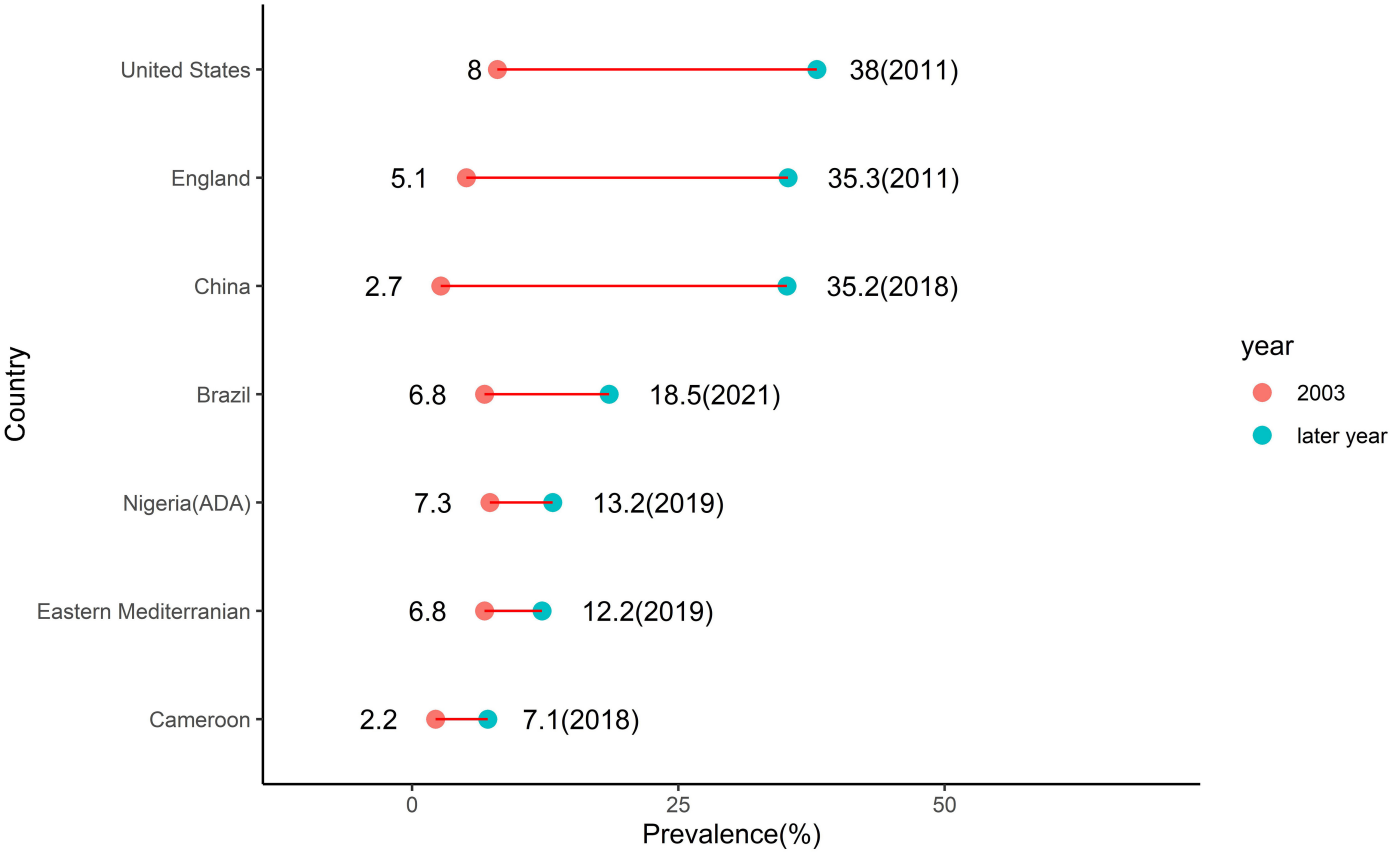
Trends in prediabetes prevalence in selected countries.

The higher pooled crude prevalence of prediabetes under the ADA criteria (13.2%) compared to pooled prevalence under the WHO criteria (10.4%) is due to the fact that the former criteria use a lower FBS threshold than the latter resulting in a higher number of individuals diagnosed with prediabetes (3). These crude prevalence rates of prediabetes in Nigeria were pooled from studies conducted between 2000 and 2019. The 2003 IDF estimate of 7.3% of prediabetes prevalence in Nigeria was pooled from two studies conducted in neighboring Cameroon in 1997 and neighboring Ghana in 2002 (8). Thus, the IDF estimate might not truly represent the burden of prediabetes in Nigeria at the time. Assuming the estimates are an accurate description of the prediabetes burden in Nigeria, the prevalence of prediabetes in the country has thus increased by 80% using ADA criteria and by 43% using WHO criteria. This rising trend of prediabetes burden is a global phenomenon. It is observed in neighboring Cameroon where in 2003 the IDF reported estimate of 2.2% increased by more than three times to 7.1% in 2018 (14). In the Eastern Mediterranean region where the IDF reported prevalence of 6.8% in 2003 almost doubled to 12.2% in 2019 (22). In Brazil where the IDF reported prevalence of 6.8% in 2003 almost tripled to 18.5% in 2021 (23). In Mainland China where the IDF reported prevalence of 2.7% in 2003 increased almost 13 times to 35.2% in 2018 (24). In England where the IDF reported prevalence of 5.1% in 2003 increased almost seven times to 35.3% in 2011 (25). In the United States where the IDF reported prevalence of 8% in 2003 increased almost five times to 38% in 2011 (26) (Figure 8).

Putting the burden of prediabetes in Nigeria in the African perspective, the number of individuals living with prediabetes in the country under the ADA criteria (15.8 million) is 35% of the whole number of adult Africans (45.3 million) with IGT as estimated in 2019 (9). This means one in every three Africans with prediabetes is a Nigerian.

Considering the reported overall type II diabetes prevalence of 5.7% in Nigeria (27, 28), the overall prevalence of dysglycemia in the country ranged from 16.1% under the WHO criteria to 18.9% under the ADA criteria. This means about one in every five adult Nigerians have dysglycemia. These individuals are at high risk of developing cardiovascular complications. This will add to the cardiovascular disease burden in the country further pressurizing the overstretched healthcare system.

Analysis of heterogeneity in this analysis reveals different behaviors for the two measures of heterogeneity: *I*^2^ and tau^2^. The former is known to be sensitive to the size of studies, i.e., if the included studies are fairly large then the sampling error will be close to zero and *I*^2^ as a ratio will approach 100% (29). To measure a prediabetes prevalence of 13% found in this study with a precision of 0.05 and 95% confidence interval, the minimum sample size required is 174 (13). The studies included in this meta-analysis are, by the selection criteria, relatively large with sample sizes displaying an interquartile range of 334. Consequently, *I*^2^ might be closer to 100% even if the between-study heterogeneity is not substantial. This is likely the case as meta-regression reduced the tau^2^ by almost 98% whereas reduction in *I*^2^ was less pronounced, though equally significant. The prediction intervals for models under ADA and WHO criteria included 0 in their lower bounds. This means that among a population of highly heterogeneous studies on prediabetes conducted or to be conducted in Nigeria, a subset of studies conducted in specific age groups, regions, and settlements will find a prevalence rate of 0. The respective highest possible prevalence rates are 52 and 46% for ADA and WHO criteria.

The similar prevalence of prediabetes in women compared to men in Nigeria found in this analysis is similar to what was found in a pooled estimate of IFG prevalence rates in the country (27). This might indicate a tendency for closure of the gap in the prevalence rates of type II diabetes between the two sexes. The reasons for this tendency might be due to the fact that women in Nigeria have higher rates of generalized obesity (BMI) (30) and physical inactivity (31). A similar equal prevalence of prediabetes in women and men was found in China (24). In Brazil, however, women were found to have a higher prevalence of prediabetes (23). However, in England and the United States, men have a higher prevalence of prediabetes than women (25, 26).

The similar prediabetes prevalence between rural and urban settlements is unexpected. This is because urban residents are less physically active due to access to motorized transport, clerical and mechanized work activity, and access to work-saving devices even for home-related activities like cooking and laundry. Access to energy-dense foods like refined sugar and saturated fats is also higher in urban settlements (32). Consequently, diabetes prevalence is higher among urban than rural residents in the country as found in a recent meta-analysis (33). The relatively high prevalence of prediabetes among rural residents might be a precursor of a future diabetic epidemic in the country.

**Figure 9:**
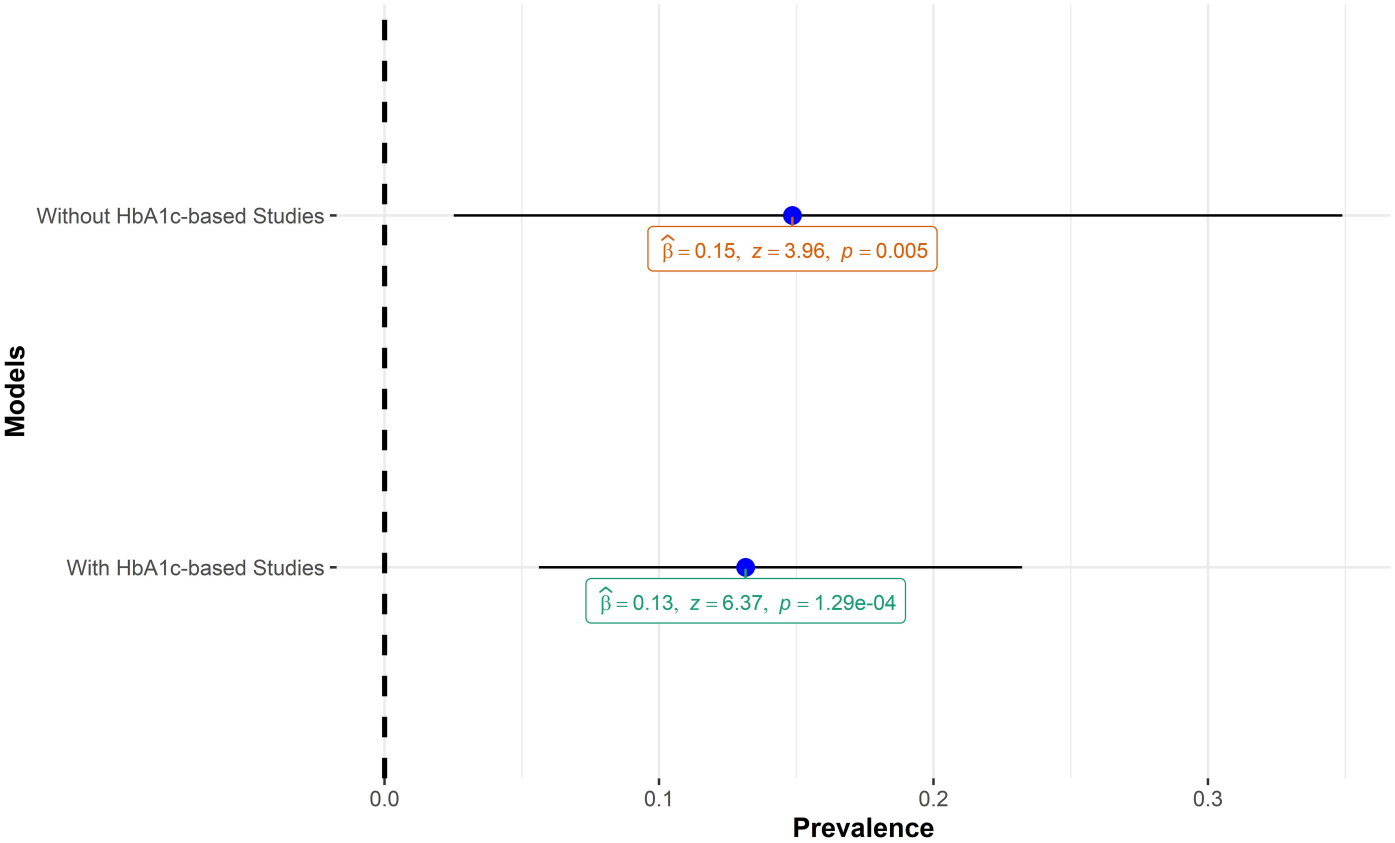
Comparison of pooled prediabetes estimates with and without HbA1c-based studies.

The pooled estimate of this meta-analysis included studies that employed HbA1c in diagnosing prediabetes. Although HbA1c is a sensitive marker for chronic hyperglycemia and useful in monitoring microvascular complications of prediabetes and diabetes (34), it is a less reliable tool for making a diagnosis of prediabetes and diabetes (35). Specifically, HbA1c values are known to be higher in individuals of African ancestry compared to Caucasians for the same level of plasma glucose levels in individuals with IGT (36) and with diabetes (37). The implication for this meta-analysis is that including HbA1c-based studies might lead to an inflated pooled estimate of prediabetes prevalence. A sensitivity analysis carried out by running the meta-analysis model with and without HbA1c-based studies showed that, in the context of this meta-analysis, including the HbA1c-based studies did not lead to inflated prevalence of prediabetes. Figure 9 shows the comparisons of the two models.

Finally, most of the included studies are of low methodological quality with subjects not representative of the general population or employing convenient sampling methods. This will have negative effect on the validity of our estimates. However, the quality appraisal of the included studies (Supplementary Table 1) might help in improving the validity as out of the 53 included studies only 15 were judged to be of good methodological quality and included in the quantitative analysis.

## Summary and Conclusion

The pooled prevalence of prediabetes in Nigeria was found to be 13.2% (95% CI: 5.6–23.2%, *I*^2^ = 98.4%) using the ADA criteria and 10.4% (95% CI: 4.3–18.9%, *I*^2^ = 99.2%) using the WHO criteria. According to the latest data by the United Nations (11), this translates to estimated 15.8 million and 12.5 million adult prediabetic individuals in Nigeria using the ADA and WHO criteria, respectively. The prevalence rates for women and men were similar at 12.1% (95% CI: 5–21%). The pooled prevalence rates for urban and rural settlements were also similar at 9% (95% CI: 2–22%). In conclusion, the prevalence of prediabetes in Nigeria was almost two times higher than the 7.3% estimate by the International Diabetes Federation in 2003. The similar rates of prediabetes between men and women and between urban and rural settlements points toward narrowing of cardiovascular risk burden between the two sexes and the two settlements. This represents higher future cardiovascular disease burden in the country further pressurizing the overstretched healthcare system.

## Data Availability Statement

The data and R markdown script used in this study are available in the GitHub repository: musabashir34/prediabetes_burden_in_Nigeria.

## Author Contributions

AIY was responsible for the conceptualization of the research. Screening of the titles and abstracts was done by MB and IM. Qualitative analysis of the selected studies was done by MM and AHY. Statistical analysis was done by MB. All authors contributed to the discussion and gave their approval for the final version of the manuscript.

## Conflict of Interest

The authors declare that the research was conducted in the absence of any commercial or financial relationships that could be construed as a potential conflict of interest.

## Publisher's Note

All claims expressed in this article are solely those of the authors and do not necessarily represent those of their affiliated organizations, or those of the publisher, the editors and the reviewers. Any product that may be evaluated in this article, or claim that may be made by its manufacturer, is not guaranteed or endorsed by the publisher.
