# Supplementary material for: Prediabetes Burden in Nigeria: A Systematic Review and Meta-Analysis
Source: Front Public Health. 2021 Dec 23;9:762429. doi: 10.3389/fpubh.2021.762429 (PMC8733165; doi:10.3389/fpubh.2021.762429)
Supplement: Supplementary file 1 [file Table_1.docx]

Supplementary Table 1: Quality Appraisal of the Selected Studies

| s/n | First Author | Study Year | Settlement | State | Region |  | Items Scores | | | | | |  | Quality |
| --- | --- | --- | --- | --- | --- | --- | --- | --- | --- | --- | --- | --- | --- | --- |
|  |  |  |  |  |  |  | sample frame | sampling | Sample size | subjects and setting | valid methods | reliable methods | Total Score |  |
| 1 | Abubakar(1) | 2012 | urban | kano | north_west | 684 | 2 | 2 | 1 | 1 | 1 | 1 | 8 | Good |
| 2 | Adediran(2) | 2012 | urban+rural | abuja | north_central | 677 | 0 | 0 | 1 | 1 | 1 | 1 | 4 | Poor |
| 3 | Adegoke(3) | 2017 | urban | osun | south_west | 220 | 0 | 0 | 1 | 1 | 1 | 1 | 4 | Poor |
| 4 | Akinshipe(4) | 2019 | mixed | edo | south_south | 352 | 2 | 2 | 1 | 1 | 1 | 1 | 8 | Good |
| 5 | Akintunde(5) | 2017 | urban | oyo | south_west | 200 | 0 | 0 | 1 | 1 | 1 | 1 | 4 | Poor |
| 6 | Akinwusi(6) | 2011 | urban | osun | south_west | 259 | 2 | 2 | 1 | 1 | 0 | 0 | 6 | Good |
| 7 | Aladeniyi(7) | 2017 | urban | ondo | south_west | 4828 | 0 | 0 | 1 | 1 | 0 | 0 | 2 | Poor |
| 8 | Alikor(8) | 2015 | rural | rivers | south_south | 300 | 2 | 0 | 1 | 1 | 1 | 1 | 6 | Good |
| 9 | Aniekwensi(9) | 2010 | urban | jos | north_central | 709 | 2 | 2 | 1 | 1 | 1 | 1 | 8 | Good |
| 10 | Anyasodor (10) | 2017 | urban | delta | south_south | 433 | 2 | 2 | 1 | 1 | 1 | 1 | 8 | Good |
| 11 | Ayogu(11) | 2019 | rural | anambra | south_east | 493 | 0 | 0 | 1 | 1 | 0 | 0 | 2 | Poor |
| 12 | Bakari (12) | 2004 | urban | kaduna | north_west | 39 | 0 | 0 | 0 | 1 | 1 | 1 | 3 | Poor |
| 13 | Casmir(13) | 2018 | urban | lagos | south_west | 293 | 0 | 0 | 1 | 1 | 1 | 1 | 4 | Poor |
| 14 | Chuhwak(14) | 2019 | rural | pleateu | north_central | 840 | 0 | 0 | 1 | 1 | 1 | 1 | 4 | Poor |
| 15 | Dahiru(15) | 2008 | rural | kaduna | north_west | 199 | 2 | 2 | 1 | 1 | 1 | 1 | 8 | Good |
| 16 | Dokunmu(16) | 2018 | urban | ogun | south_west | 182 | 0 | 0 | 1 | 1 | 0 | 0 | 2 | Poor |
| 17 | Ejike(17) | 2015 | urban | abia | south_east | 365 | 0 | 0 | 1 | 1 | 0 | 0 | 2 | Poor |
| 18 | Ejike(18) | 2019 | urban | ebonyi | south_east | 720 | 2 | 2 | 1 | 1 | 0 | 0 | 6 | Good |
| 19 | Enang (19) | 2014 | urban | calabar | south_south | 1134 | 2 | 2 | 1 | 1 | 1 | 1 | 8 | Good |
| 20 | Eru(20) | 2018 | urban | benue | north_central | 5120 | 2 | 2 | 1 | 1 | 0 | 0 | 6 | Good |
| 21 | Ezeala-Adikaibe(21) | 2018 | urban | enugu | south_east | 605 | 0 | 0 | 1 | 1 | 1 | 1 | 4 | Poor |
| 22 | Gwarzo(22) | 2020 | urban | kano | north_west | 148 | 0 | 0 | 1 | 1 | 0 | 0 | 2 | Poor |
| 23 | Lawal(24) | 2018 | urban | kaduna | north_west | 400 | 2 | 2 | 1 | 1 | 1 | 1 | 8 | Good |
| 24 | Lawal_a(25) | 2019 | urban | bauchi | north_east | 172 | 0 | 0 | 1 | 1 | 0 | 0 | 2 | Poor |
| 25 | Martins(26) | 2017 | urban | ibadan | south_west | 300 | 0 | 0 | 1 | 1 | 1 | 1 | 4 | Poor |
| 26 | Nwatu(27) | 2013 | rural | enugu | south_east | 771 | 2 | 2 | 1 | 1 | 0 | 0 | 6 | Good |
| 27 | Nwatu(28) | 2020 | rural | enugu | south_east | 538 | 0 | 0 | 1 | 1 | 0 | 0 | 2 | Poor |
| 28 | Nyenwe(29) | 2000 | urban | port harcourt | south_south | 502 | 2 | 2 | 1 | 1 | 1 | 1 | 8 | Good |
| 29 | Ogbu(30) | 2012 | urban | imo | south_east | 1012 | 0 | 0 | 1 | 1 | 1 | 1 | 4 | Poor |
| 30 | Oguoma(31) | 2015 | urban+rural | mixed | south_south, south_east, south_west & north_central | 2447 | 0 | 0 | 1 | 1 | 0 | 0 | 2 | Poor |
| 31 | Ojewale(32) | 2013 | urban | ibadan | south_west | 301 | 0 | 0 | 1 | 1 | 0 | 0 | 2 | Poor |
| 32 | Oladapo(33) | 2005 | rural | oyo | south_west | 2000 | 2 | 2 | 1 | 1 | 1 | 1 | 8 | Good |
| 33 | Olatona(34) | 2018 | urban | lagos | south_west | 503 | 0 | 0 | 1 | 1 | 1 | 1 | 4 | Poor |
| 34 | Olatunbosun(35) | 1998 | urban | ibadan | south_west | 998 | 0 | 0 | 1 | 1 | 1 | 1 | 4 | Poor |
| 35 | Oluyombo(36) | 2014 | urban | ekiti | south_west | 750 | 0 | 0 | 1 | 1 | 0 | 0 | 2 | Poor |
| 36 | Onuoha(37) | 2016 | urban | enugu | south_east | 39 | 2 | 2 | 0 | 1 | 0 | 0 | 5 | Poor |
| 37 | Onyemelukwe(38) | 2020 | urban | kaduna | north_west | 217 | 0 | 0 | 1 | 1 | 0 | 0 | 2 | Poor |
| 38 | Ozomma(39) | 2017 | urban | anambra | south_east | 277 | 0 | 0 | 1 | 1 | 1 | 1 | 4 | Poor |
| 39 | Popoola(40) | 2015 | urban | ibadan | south_west | 371 | 0 | 0 | 1 | 1 | 1 | 1 | 4 | Poor |
| 40 | Sabir(41) | 2008 | rural | sokoto | north_west | 423 | 2 | 2 | 1 | 1 | 1 | 1 | 8 | Good |
| 41 | Sani(42) | 2010 | urban | katsina | north_west | 321 | 0 | 0 | 1 | 1 | 0 | 0 | 2 | Poor |
| 42 | Sedodo(43) | 2019 | urban | ogun | south_west | 300 | 0 | 0 | 1 | 1 | 0 | 0 | 2 | Poor |
| 43 | Tanimu(44) | 2019 | urban | nasarawa | north_central | 168 | 0 | 0 | 1 | 1 | 0 | 0 | 2 | Poor |
| 44 | Umuerri(45) | 2020 | urban | delta | south_south | 159 | 0 | 0 | 1 | 1 | 0 | 0 | 2 | Poor |
| 45 | Uwanuruochi(46) | 2013 | urban | abia | south_east | 299 | 0 | 0 | 1 | 1 | 1 | 1 | 4 | Poor |
| 46 | Wahab(47) | 2008 | urban | katsina | north_west | 321 | 0 | 0 | 1 | 1 | 1 | 1 | 4 | Poor |
| 47 | Essiet(48) | 2019 | urban | delta | south-south | 420 | 0 | 0 | 1 | 1 | 1 | 1 | 4 | Poor |
| 48 | Felix(49) | 2019 | urban | abuja | north_central | 96 | 0 | 0 | 0 | 1 | 1 | 1 | 3 | Poor |
| 49 | Idemudia(50) | 2019 | urban | edo | south_south | 156 | 0 | 0 | 1 | 1 | 1 | 1 | 4 | Poor |
| 50 | Kabir(51) | 2020 | urban | jigawa | north_west | 100 | 0 | 0 | 0 | 1 | 0 | 0 | 1 | Poor |
| 51 | Nwafor(52) | 2015 | rural | rivers | south_south | 250 | 0 | 0 | 1 | 1 | 0 | 0 | 2 | Poor |
| 52 | Nwose (53) | 2015 | urban | delta | south_south | 74 | 0 | 0 | 0 | 1 | 0 | 0 | 1 | Poor |
| 53 | Mezie-Okoye(54) | 2013 | urban | anambra | south_east | 147 | 0 | 0 | 1 | 1 | 0 | 0 | 2 | Poor |

REFERENCES

1. Abubakar LY. PREVALENCE AND CORRELATES OF DIABETES MELLITUS AND GLUCOSE INTOLERANCE AMONG ADULTS IN KANO METROPOLIS. Faculty of INTERNAL MEDICINE [Internet]. 2012 [cited 2021 Jul 5]; Available from: <https://dissertation.npmcn.edu.ng/index.php/FMCP/article/view/700>

2. Adediran O, Akintunde AA, Edo AE, Opadijo OG, Araoye AM. Impact of urbanization and gender on frequency of metabolic syndrome among native Abuja settlers in Nigeria. Journal of Cardiovascular Disease Research. 2012;3(3):191–6.

3. Adegoke OA, Emma-Okon BO, Fasanya MK, Salawu AO, Tomi-Olugbodi AA. Behavioural and anthropometric risk factors for diabetes mellitus among newly admitted undergraduates in a Nigerian University. Nigerian Journal of Clinical Practice. 2017 Jan;20(10):1246.

4. Akinshipe BO, Yusuf EO, Akinshipe FO, Moronkeji MA, Nwaobi AC. Prevalence and Determinants of Pre-diabetes and Latent Tuberculosis Infection Among Apparently Healthy Adults in Three Communities in Southern Nigeria. International Journal of Immunology. 2019 Aug;7(2):23.

5. Akintunde AA, Oloyede TW. Metabolic syndrome and occupation: Any association? Prevalence among auto technicians and school teachers in South West Nigeria. Diabetes & Metabolic Syndrome: Clinical Research & Reviews. 2017 Nov;11:S223–7.

6. Akinwusi PO, Fwacp EOA-O, Fwacp WOA, Fwacs MAI, FMCOph, Fwacs MBH, et al. Cardiovascular risk factors and electrocardiographic pattern in two rural communities of Osun State in South West Nigeria. Current Research: Cardiology. 2017 Jul;4(2).

7. Aladeniyi I, Adeniyi OV, Fawole O, Adeolu M, Ter Goon D, Ajayi AI, et al. The Prevalence and Correlates of Pre-Diabetes and Diabetes Mellitus Among Public Category Workers in Akure, Nigeria. The Open Public Health Journal. 2017 Sep;10(1).

8. Alikor CA, Emem-Chioma PC. EPIDEMIOLOGY OF DIABETES AND IMPAIRED FASTING GLUCOSE IN A RURAL COMMUNITY OF NIGERIAN NIGER DELTA REGION. Nigerian Journal of Medicine: Journal of the National Association of Resident Doctors of Nigeria. 2015 Apr-Jun;24(2):114–24.

9. Aniekwensi E. A COMPARATIVE EVALUATION OF BODY MASS INDEX, WAIST CIRCUMFERENCE AND WAIST-TO-HIP RATIO AS CORRELATES OF GLUCOSE INTOLERANCE AMONG URBAN ADULTS IN JOS METROPOLIS, NIGERIA. Faculty of INTERNAL MEDICINE [Internet]. 2010 [cited 2021 Jul 5]; Available from: <https://dissertation.npmcn.edu.ng/index.php/FMCP/article/view/554>

10. Anyasodor AE, Nwose EU, Bwititi PT, Aganbi E, Richards RS, Mudiaga LI, et al. Prevalence of hyperglycemia and risk factors for orodental disease in Nigeria: Implications of opportunistic screening. Indian Journal of Dental Research: Official Publication of Indian Society for Dental Research. 2017 Sep-Oct;28(5):507–13.

11. Ayogu RNB, Nwajuaku C, Udenta EA. Components and risk factors of metabolic syndrome among rural Nigerian workers. Nigerian Medical Journal. 2019 Jan;60(2):53.

12. Bakari AG, Onyemelukwe GC. Glucose intolerance among apparently healthy Hausa-Fulani Northern Nigerians. Annals of African Medicine [Internet]. 2004 Aug [cited 2021 Jul 5];3(1). Available from: <https://www.ajol.info/index.php/aam/article/view/8298>

13. Casmir EA, Amam CM, Obianuju BO, Tim PG, David AW, Oyewole AK, et al. Prevalence of cardiometabolic risk factors among professional male longdistance bus drivers in Lagos, southwest Nigeria: A crosssectional study. Cardiovascular Journal of Africa. 2018;29(2):106–14.

14. Chuhwak EK, Okeahialam BN, Ogbonna C, Pam SD. Diabetes in elderly Nigerians: A survey of a rural area in north-central Nigeria. Journal of Medicine in the Tropics. 2019 Jan;21(2):51.

15. Dahiru T, Jibo A, Hassan AA, Mande AT. Prevalence of diabetes in a semi-urban community in Northern Nigeria. Nigerian Journal of Medicine: Journal of the National Association of Resident Doctors of Nigeria. 2008 Oct-Dec;17(4):414–6.

16. Dokunmu TM, Yakubu OF, Adebayo AH, Olasehinde GI, Chinedu SN. Cardiovascular Risk Factors in a Suburban Community in Nigeria. International Journal of Hypertension. 2018 Apr;2018:e6898527.

17. Ejike C, Uka N, Nwachukwu S. Diabetes and pre-diabetes in adult Nigerians: Prevalence, and correlations of blood glucose concentrations with measures of obesity. African Journal of Biochemistry Research. 2015 Apr;9:55–60.

18. Ejike CECC, Onyeji GN, Odoh M, Nwali CM, Ivoke FO, Ubaezuonu AV, et al. Double Jeopardy: Preponderance of Impaired Glucose Homeostasis and Overweight/Obesity Among Adult Females in Ikwo, Ebonyi State. Journal of Obesity and Chronic Diseases. 2019;03(01).

19. Enang OE, Otu AA, Essien OE, Okpara H, Fasanmade OA, Ohwovoriole AE, et al. Prevalence of dysglycemia in Calabar: A cross-sectional observational study among residents of Calabar, Nigeria. BMJ Open Diabetes Research and Care. 2014 Jun;2(1):e000032.

20. Eru DE, Eke DB, Onahinon C, Ibu J. Relationship Between Body Mass Index, And Type 2 Diabetes Mellitus Among Adult Nigerians In Makurdi, Nigeria. :7.

21. Ezeala-Adikaibe B, Mbadiwe N, Okwara C, Onodugo O, Onyekonwu C, Ijoma U, et al. Diabetes and Pre-Diabetes among Adults in an Urban Slum in South East Nigeria. Journal of Diabetes Mellitus. 2018 Jan;08:131–44.

22. Gwarzo I Mukhtar, Wali N, Ahmed Ibrahim S. Correlation of Anthropometric Indices with Fasting Blood Glucose and Blood Pressure Among University Students in Kano, Nigeria. 2020 Oct;17:128–34.

23. Humanitarian Data Exchange. Nigeria - Subnational Administrative Boundaries [Internet]. The Humanitarian Data Exchange. 2020 [cited 2021 Jul 4]. Available from: <https://data.humdata.org/dataset/nga-administrative-boundaries>

24. Lawal Y, Anumah FE, Bakari AG. Is Glycated Haemoglobin an Alternative to Diagnose Diabetes Mellitus in a Northern Nigerian Population? Annals of Medical and Health Sciences Research [Internet]. 2018 [cited 2021 Jul 5]; Available from: <https://www.amhsr.org/abstract/is-glycated-haemoglobin-an-alternative-to-diagnose-diabetes-mellitus-in-a-northern-nigerian-population-4485.html>

25. Lawal Y, Muhammad S, Dahuwa UF, Bichi I, Ahmed H. Screening for obesity and undiscovered glucose intolerance among employees of a tertiary health center in northeast Nigeria. Journal of Health Research and Reviews,India [Internet]. 2019 [cited 2021 Jul 5]; Available from: <https://www.jhrr.org/article.asp?issn=2394-2010;year=2019;volume=6;issue=3;spage=107;epage=113;aulast=Lawal>

26. Martins SO, Folasire OF, Irabor AE. PREVALENCE AND PREDICTORS OF PREDIABETES AMONG ADMINISTRATIVE STAFF OF A TERTIARY HEALTH CENTRE, SOUTHWESTERN NIGERIA. Annals of Ibadan Postgraduate Medicine [Internet]. 2017 Dec [cited 2021 Jul 5];15(2):114–23. Available from: <https://www.ncbi.nlm.nih.gov/pmc/articles/PMC5846173/>

27. Nwatu CB, Young EE, Onyenekwe BM, Ezike CH, Ugwu ET, Obi PC. Association of short sleep duration with cardiometabolic risk factors in a population of rural Nigerian women: A cross-sectional study. International Journal of Medicine and Health Development. 2020 Jan;25(2):120.

28. Nwatu CB, Young EE, Okwara CC, Okoli CE, Obi PC, Anyim OB, et al. Concurrent Prediabetes and Prehypertension in a Rural Community in South East Nigeria. Journal of Advances in Medicine and Medical Research. 2017 Jun;1–0.

29. Nyenwe EA, Odia OJ, Ihekwaba AE, Ojule A, Babatunde S. Type 2 diabetes in adult Nigerians: A study of its prevalence and risk factors in Port Harcourt, Nigeria. Diabetes Research and Clinical Practice. 2003 Dec;62(3):177–85.

30. Ogbu ISI, Azodo EC, Chinwuba AU. Prevalence of pre-diabetes and unreported diabetes mellitus in population aged 45 years and above in Owerri Municipality, Imo State Nigeria. International Journal of Medicine and Health Development. 2012;17(2):31–8.

31. Oguoma VM, Nwose EU, Ulasi II, Akintunde AA, Chukwukelu EE, Bwititi PT, et al. Cardiovascular disease risk factors in a Nigerian population with impaired fasting blood glucose level and diabetes mellitus. BMC Public Health. 2017 Jan;17(1):36.

32. Ojewale LY, Adejumo PO. Relationship between diabetes risk factors and fasting blood glucose among civil servants in Ibadan, Nigeria. International Journal of Diabetes in Developing Countries. 2014 Sep;34(3):139–43.

33. Oladapo O, Falase A, Salako L, Sodiq O, Shoyinka K, Adedapo K. A prevalence of cardiometabolic risk factors among a rural Yoruba south-western Nigerian population: A population-based survey. Cardiovascular Journal of Africa [Internet]. 2010 Feb [cited 2021 Jul 21];21(1):26–31. Available from: <https://www.ncbi.nlm.nih.gov/pmc/articles/PMC3721297/>

34. Olatona FA, Onabanjo OO, Ugbaja RN, Nnoaham KE, Adelekan DA. Dietary habits and metabolic risk factors for non-communicable diseases in a university undergraduate population. Journal of Health, Population and Nutrition. 2018 Aug;37(1):21.

35. Olatunbosun ST, Ojo PO, Fineberg NS, Bella AF. Prevalence of diabetes mellitus and impaired glucose tolerance in a group of urban adults in Nigeria. Journal of the National Medical Association [Internet]. 1998 May [cited 2021 Aug 1];90(5):293–301. Available from: <https://www.ncbi.nlm.nih.gov/pmc/articles/PMC2608340/>

36. Oluyombo R, Olamoyegun MA, Olaifa O, Iwuala SO, Babatunde OA. Cardiovascular risk factors in semi-urban communities in southwest Nigeria: Patterns and prevalence. Journal of Epidemiology and Global Health. 2015 Jun;5(2):167–74.

37. Onuoha N, Am O, Pe E, Ud O. Anthropometric status, fasting blood sugar, nutrient intake and energy balance of traders in a market population in Nsukka, Nigeria. Integrative Food, Nutrition and Metabolism. 2016;4(1).

38. Onyemelukwe OU, Mamza AA, Suleiman YK, Iyanda MA, Bello-Ovosi B, Bansi KI, et al. Prevalence of Pre-Diabetes, Diabetes and Associated Cardiovascular Risk Amongst Healthcare Workers in Ahmadu Bello University Teaching Hospital (ABUTH), Zaria using Glycated Haemoglobin. West African Journal of Medicine. 2020 Apr-Jun;37(2):91–9.

39. Ozomma OP, J. u E, S. c M, E. c O, R. a A, I. p E, et al. Prevalence of Prediabetes and the Associated Risk of Kidney Disease in Apparently Healthy Subjects in Nnewi, Anambra State, Nigeria. Journal of Diseases [Internet]. 2017 [cited 2021 Jul 5];4(2):27–33. Available from: <https://econpapers.repec.org/article/pkpjoudis/2017_3ap_3a27-33.htm>

40. Popoola OO. Prevalence of Obesity, Hyperglycemia and Dyslipidemia and Associated Factors among Senior Staff of the Univeristy College Hospital Ibadan [Internet] [PhD thesis]. AFRICAN DIGITAL HEALTH REPOSITORY PROJECT; 2015. Available from: <http://adhlui.com.ui.edu.ng/jspui/bitstream/123456789/1139/1/UI_Dissertation_Popoola_OO_Prevalence_2015.pdf>

41. Sabir AA. GLUCOSE TOLERANCE AMONG RURAL AND URBAN FULANI OF NORTHERN NIGERIA. Faculty of INTERNAL MEDICINE [Internet]. 2008 [cited 2021 Jul 5]; Available from: <http://www.dissertation.npmcn.edu.ng/index.php/FMCP/article/view/520>

42. Sani MU, Wahab KW, Yusuf BO, Gbadamosi M, Johnson OV, Gbadamosi A. Modifiable cardiovascular risk factors among apparently healthy adult Nigerian population - a cross sectional study. BMC Research Notes. 2010;3(1):11.

43. Sedodo NS, Abosede OP, Ilori OA, Adenekan MK, Idowu OM, Nupo AO, et al. Assessment of Fasting Blood Glucose Level of Undergraduates in Abeokuta Ogun State Nigeria. Archives of Current Research International. 2020 Jul;42–9.

44. Tanimu H, Paul AD. Investigation of the Occurrence of Abnormal Oral Glucose Tolerance (OGT) Level among People of Nasarawa State Polytechnic, Lafia. Agricultural Research & Technology: Open Access Journal. 2019 Apr;10(4):001–5.

45. Umuerri EM. Risk assessment of type 2 diabetes and validation of a non-invasive risk-prediction tool among women in an urban community in Delta State, Nigeria. Journal of Community Medicine and Primary Health Care. 2020 Apr;32(1):47–58.

46. Uwanuruochi K, Ukpabi O, Onwuta C, Onwubere B, Anisiuba B, Michael F. Cardiovascular Risk Factors in Adult Staff of Federal Medical Centre, Umuahia:a Comparison with other Nigerian Studies. West African journal of medicine. 2013 Oct;32:243–7.

47. Wahab K, Sani M, Gbadamosi M, Yandutse M. Frequency and determinants of the metabolic syndrome in apparently healthy adult Nigerians. Tropical doctor. 2008 Nov;38:224–6.

48. Essiet DF, Osadolor HB. Prevalence of pre-diabetes and undiagnosed diabetes mellitus among adults in the Warri Metropolis, Nigeria. New Zealand Journal of Medical Laboratory Science. 73(1):6–10.

49. Felix S. Evaluation of Haematological Parameters among Pre- Diabetic and Diabetic Subjects in Urban Setting. International Journal of Science and Research Methodology. 2019 Jun;

50. Idemudia JO, Atoe K. A Pilot Assessment of Dyslipidaemia and Risk of Arteriosclerosis among Men in Oredo Local Government Area of Edo State, Nigeria. JOURNAL OF RESEARCH IN BASIC AND CLINICAL SCIENCES [Internet]. 2019 Dec [cited 2021 Aug 3];1(3):243–52. Available from: <https://jrbcs.org/index.php/jrbcs/article/view/62>

51. Kabir, N Aujara, I, Ibrahim, S. Prevalence of prediabetes and its associated risk factors among staff and students of Federal University Dutse.2020 BJMLS, 5(1): 1–9.

52. Nwafor A, Mmom FC, Obia O, Obiandu C, Hart VO, Chinko BC. Relationship between Blood Pressure, Blood Glucose and Body Mass Index and Coexisting Prehypertension and Prediabetes among Rural Adults in Niger Delta Region, Nigeria. Journal of Advances in Medicine and Medical Research. 2015 Jul;1–2.

53. Nwose EU, Oguoma VM, Bwititi PT, Richards RS. Metabolic Syndrome and Prediabetes in Ndokwa Community of Nigeria: Preliminary Study. North American Journal of Medical Sciences. 2015 Feb;7(2):53–8.

54. Mezie-Okoye M. Diabetes in older adults: Experience from a rural community in south-east Nigeria. African Journal of Diabetes Medicine. 2013 Nov;Vol 21:45–9.
